# HCV Core/NS3 Protein Immunization with “N-Terminal Heat Shock gp96 Protein (rNT (gp96))” Induced Strong and Sustained Th1-Type Cytokines in Immunized Mice

**DOI:** 10.3390/vaccines9030215

**Published:** 2021-03-03

**Authors:** Zamaneh Hajikhezri, Farzin Roohvand, Monireh Maleki, Shohreh Shahmahmoodi, Ali Akbar Amirzargar, Abolfazl Keshavarz, Negar Seyed, Mohammad Farahmand, Katayoun Samimi-Rad

**Affiliations:** 1 Department of Virology, School of Public Health, Tehran University of Medical Sciences, Tehran 1449614535, Iran; z-hajikhezri@razi.tums.ac.ir (Z.H.); Shahmahmoodi@tums.ac.ir (S.S.); a-keshavarz@farabi.tums.ac.ir (A.K.); Farahmandm@outlook.com (M.F.); 2 Department of Virology, Pasteur Institute of Iran, Tehran 1316943551, Iran; farzin.roohvand2@gmail.com; 3 Department of Clinical Biochemistry, Faculty of Medical Sciences, Islamic Azad University of Tehran, Tehran 1477893855, Iran; Monireh.maleki@gmail.com; 4 Food Microbiology Research Center, Tehran University of Medical Sciences, Tehran 1449614535, Iran; 5 Molecular Immunology Research Center, Tehran University of Medical Sciences, Tehran 1449614535, Iran; amirzara@sina.tums.ac.ir; 6 Immunogenetic Laboratory, Department of Immunology, Faculty of Medicine, Tehran University of Medical Sciences, Tehran 1449614535, Iran; 7 Department of Immunotherapy and Leishmania Vaccine Research, Pasteur Institute of Iran, Tehran 1316943551, Iran; negarse@gmail.com

**Keywords:** Hepatitis C virus, rNT (gp96), vaccine, Core-Ns3, prime-boost

## Abstract

Feeble cellular responses induced by T cell-based vaccines are a major challenge for the development of an effective vaccine against Hepatitis C virus (HCV) infection. To address this challenge, the potential of N-terminal fragment of gp96 heat shock protein (rNT (gp96) as an adjuvant was evaluated and compared to that of the CpG (as a recognized Th1-type adjuvant) in the formulation of HCV core/NS3 antigens in three immunization strategies of protein/protein, DNA/DNA, and DNA/protein. Immunized mice were evaluated for elicited immune responses in week 3 (W3) and 11 post-immunizations. Our results demonstrated that the protein (subunit) vaccine formulated with rNT (gp96) in protein/protein strategy (core/NS3 + gp96) was significantly more efficient than CpG oligodeoxynucleotides (CpG ODN) formulation and all other immunization strategies in the induction of Th1-type cytokines. This group of mice (core/NS3 + gp96) also elicited a high level of anti-Core-NS3 total immunoglobulin G (IgG) with dominant IgG2a isotype at W3. Thus, the co-administration of recombinant NT (gp96) protein with rHCV proteins might be a promising approach in the formulation of HCV subunit vaccine candidates for induction of high levels of Th1 cytokines and humoral responses.

## 1. Introduction

Infection with Hepatitis C virus (HCV) turns to chronic hepatitis in the majority of the infected individuals that might result in liver cirrhosis or malignancies in time. Although recently invented direct-acting antiviral drugs (DAAs) can cure a high percentage of chronic hepatitis C (CHC) patients [1], only a small fraction (less than 10%) have access to the treatments, which is partly due to the high cost and limitations in the screening of the patients (5–20% screening rate of the estimated 71 million infected people) [2,3,4,5]. Indeed, recent reports revealed that the number of newly infected HCV cases (1.75 million annually) exceeds that of the treatment rate [2]. Moreover, many DAA-treated CHC patients with cirrhotic liver might still develop liver cancer, even after HCV clearance [6]. Therefore, along with treating patients with DAAs, an effective vaccine is absolutely required to control HCV infection worldwide.

HCV genome encodes for several structural (core and E1, E2) and non-structural proteins (p7 to NS5) [7]. The lack of efficient cell culturing systems and intrinsic dangers encountered with large HCV propagation directed the vaccine development studies to the use of HCV proteins in novel vaccine platforms [8]. In general, two strategies targeting either the generation of neutralizing antibodies (Nabs; directed against E2 protein) or T cell cellular responses (directed against the core and NS3–NS5 proteins) are addressed for HCV vaccine development [9,10]. However, reports on resolution of acute HCV infection even before the establishment of Nabs [11] or in antibody-deficient patients (such as those with hypogammaglobinemia) [12], and the limited cross genotype reactivity of the elicited Nabs among different HCV types/subtypes (due to hypervariable regions in E2) [13] diminished the importance of Nabs in vaccine development studies. In contrast, data from studies on HCV T cell vaccines showed the importance of cellular responses such as the establishment of persistent HCV infection where CD4+ and CD8+ T cells from HCV challenged chimpanzees were depleted and critical association between HCV clearance and specific class I and class II human leukocyte antigens (HLAs) which present HCV peptides to CD4+ and CD8+ T cells, respectively [10,14]. Among HCV proteins, core and NS3, which are highly conserved and contain well-characterized T cell determinants associated with the clearance of HCV infection, have been the target of many vaccine formulation studies in various platforms for induction of Th1-balance cellular responses [15,16]. However, the induction of robust immune responses against isolated proteins of pathogens requires the use of proper adjuvant formulations [17]. Notably, prior studies indicated the necessity of Th1-adjuvant formulations to induce cellular responses against HCV NS proteins [18]. This is even the case with “DNA vaccine” approaches that usually induce Th1-biased immunity but in lower levels [19]. However, some approaches such as incorporating immune-stimulatory DNA sequences (such as CpG, Cytokines, and Heat shock proteins) and/or heterologous prime-boost vaccination has been demonstrated to augment immune responses induced by DNA vaccines, including those encoding HCV core protein [20,21,22]. The heat shock protein (HSP) gp96, particularly its N-terminal domain “NT (gp96)”, and CPG ODNs are among well-known human-compatible adjuvants and immune-stimulants that are used in the formulation of both DNA and protein-based vaccines. The HSP gp96 plays chaperoning roles in innate and adaptive immune responses [23] and has been used in the formulation of several viral proteins from Hepatitis B virus, H1N1 Influenza, and porcine reproduction and respiratory syndrome virus as a vaccine adjuvant [24,25,26]. To date, no study has addressed the use of gp96 for the formulation of HCV proteins as vaccine adjuvants, and the only report belongs to a fusion of NT (gp96) sequence to a multiepitope HCV DNA vaccine [27]. The other adjuvant, CpG ODNs, is known for enhancing the Th1-biased immune responses [20]. Several studies reported the utilization of CpG ODNs in the induction of immune responses against HCV proteins (including core and NS3) and thus might be a proper component to compare the adjuvant effect of other immune stimulants in the formulation of HCV antigens (Ags) [28,29]. Although CpG ODNs were used in the formulation of HCV core and NS3 alone or in combination with other HCV proteins to our knowledge, no prior study addressed its adjuvant effect in combination with both HCV core and NS3 in a single Ag formulation.

In the present study, we investigated and compared the adjuvant effects of NT (gp96) and CpG ODNs in the formulation of both recombinant proteins and DNA vaccines encoding HCV core and NS3 Ags (core/NS3) in various protein/protein, DNA/DNA, and DNA/protein immunization regimens in mice. We also studied the long-term efficacy of the vaccine candidates in all immunized mice groups.

## 2. Materials and Methods

### 2.1. Constructs of the Expression Vector and Purification of Proteins

#### 2.1.1. Construction of Recombinant Expression Vectors

**Figure 1:**
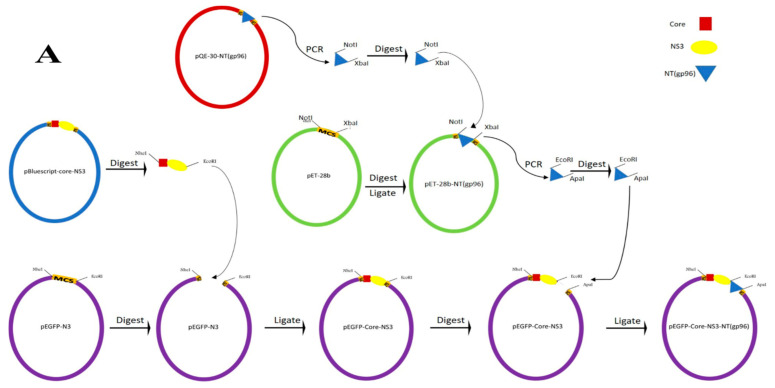

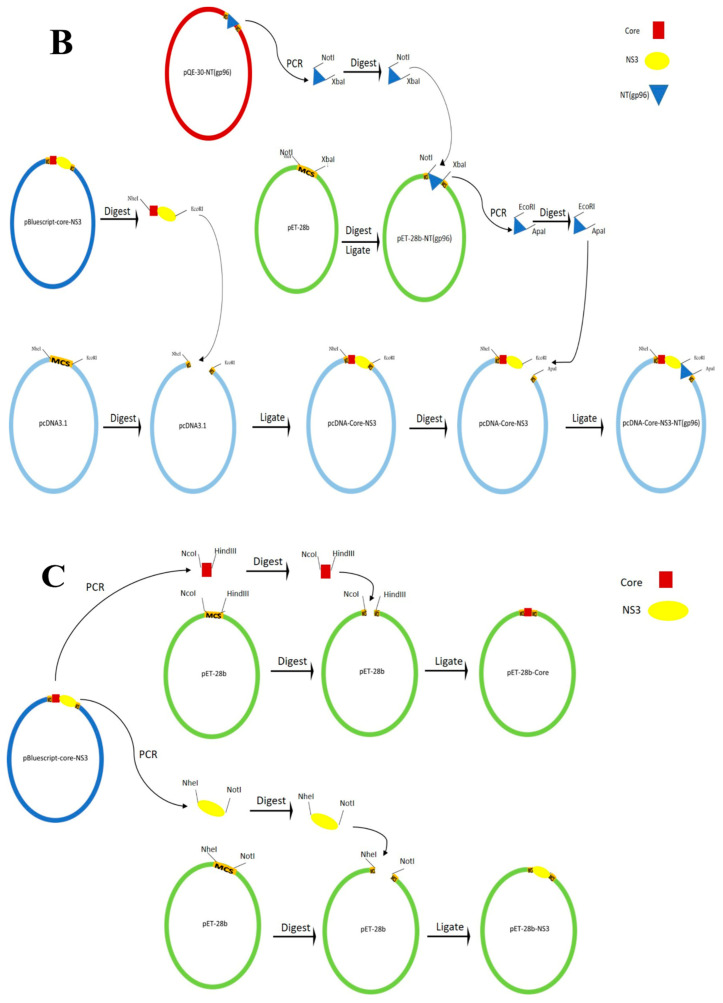
Schematic representation for the construction of the vectors. (**A**) pEGFP-Core-NS3-NT (gp96), (**B**) pcDNA-Core-NS3, pcDNA-Core-NS3-NT (gp96), and (**C**) pET-28b-Core, pET-28b-NS3.

The constructions of Core-NS3 encoding vectors are illustrated in Figure 1, and used primers are presented in Table 1. In brief, the amino acids 1–120 and 1167–1457 corresponding to the core and NS3 proteins of HCV-1a strain H77 (GenBank accession no: AF011751), respectively, were synthesized as a single Core-NS3 fragment and cloned in-frame into pBluescript II SK(+) vector to construct the pBluescript-Core-NS3 plasmid (Biomatik Corporation, Kitchener, ON, Canada). The plasmids pEGFP-Core-NS3 and pcDNA-Core-NS3 were generated by subcloning of the Core-NS3 fragment from the pBluescript-Core-NS3 plasmid into pEGFP-N3 (Clontech, Palo Alto, CA, USA) and pcDNA 3.1(+) (Invitrogen, Berlin, Germany) vectors using *Nhe*I and *EcoR*I restriction sites (Figure 1A,B). Subsequently, the pQE-30 vector harboring the heat shock protein (gp96) [27] was kindly provided by Dr. Sima Rafati (Pasteur Institute of Iran, Department of immunotherapy and Leishmania vaccine research, Iran) and used as a PCR template to amplify nucleotides 1 to 1014 of gp96 (designated as NT (gp96)) using Kgp1/2 primer pairs. The PCR amplified NT (gp96) fragment was cloned into the *Not*I and *Xba*I sites of the pET-28b vector (Novagen, Madison, WI, USA) to construct pET-28b-NT (gp96). Subsequently, to prepare pEGFP-Core-NS3-NT (gp96), the NT (gp96) sequence was amplified from pET-28b-NT (gp96) using KZ1/2 primer pairs and inserted into the *EcoR*I and *Apa*I restriction sites of pEGFP-N3 (Invitrogen) (Figure 1A). The same procedure was performed to prepare pcDNA-Core-NS3-NT (gp96) with the only difference in reverse primer (KZ3), which had a stop codon (bold) in addition to the *Apa*I restriction site (Figure 1B). The pcDNA-Core-NS3 and pcDNA-Core-NS3-NT (gp96) were purified by ion-exchange chromatography with Endo Free Plasmid Giga kit (QIAGEN, Hilden, Germany). The construction of the two expression vectors pET-28b-Core and pET-28b-NS3 was also started by amplification of core and NS3 fragments using the pBluescript-Core-NS3 vector as a template employing ZC1/2 and ZN1/2 primers, respectively (Figure 1C and Table 1). The precision and accuracy of the DNA constructs were confirmed by restriction analysis and DNA sequencing. Molecular and cloning procedures were based on routine protocols [30] and/or manufacturer’s recommendations.

#### 2.1.2. Expression and Purification of the Recombinant Proteins

The expression of the Core-NS3 and Core-NS3-NT (gp96) proteins was tested in the COS-7 cell line. 5 × 10^4^ COS-7 cells per well were seeded on a four-well plate (Greiner, Pleidelsheim, Germany) and transfected when the cells reached a confluency of 75%. Five μg of pEGFP-Core-NS3, pEGFP-Core-NS3-NT (gp96), pEGFP-N3 (positive control), and pcDNA3.1 (negative control) plasmids were transfected using linear polyethyleneimine (MW = 25 kDa) (10 µM; Poly Sciences, Hirschberg an der Bergstraße, Germany) as described previously [31]. At 24 h post-transfection, the expression of proteins was confirmed by direct observation of the EGFP signal under a fluorescence microscope (Nikon E200, Melville, NY, USA). To produce the recombinant NS3, core, and NT (gp96) proteins, the bacterial cultures (transformed BL21 DES for pET-28b-NS3 and pET-28b-Core and *E. coli* M15 for pQE30-NT (gp96)) were grown in 2 xyt broth (16 g Trypton, 10 g yeast extract and 5 g NaCl per liter, pH 7.0–7.2) to an optical density of 0.7–0.8 at 600 nm. The protein expression was induced with 1 mM IPTG at 16 °C for rNS3 and 37 °C for core and NT (gp96) proteins. The cell pellets were collected and stored at −20 °C until used for protein purification. The cells were lysed by lysozyme digestion followed by sonication. The protein samples were analyzed by SDS-PAGE in 10% (*w*/*v*) polyacrylamide gel-based on standard protocols (Sambrook and Russell, 2006). The protein bands were transferred onto a polyvinylidene difluoride (PVDF) membrane using a Bio-Rad wet blotting system, and the membrane was incubated with blocking solution (TBS 10× (pH 7.4), 0.1% tween 20, 2.5% BSA) overnight. After washing steps, the membrane was treated with mouse anti-His monoclonal antibody (1:15,000, Qiagen, Hilden, Germany) and horseradish peroxidase-conjugated goat anti-mouse IgG antibody (3:8000; Southern Biotech, Birmingham, AL, USA) as the first and second antibodies, respectively. Immunoreactive bands were detected using an enhanced chemiluminescence (ECL) kit (ClarityTM Western ECL Substrates, Bio-Rad, Hercules, CA, USA), and light emission was visualized by exposing the membrane to a Hyper Film ECL (GE-Amersham, Amersham, UK). The 6xHis-tag encoding recombinant core, NS3 and NT (gp96) proteins were purified by affinity chromatography on a Ni-NTA superflow column. The purification was performed under native conditions according to the manufacturer’s instructions (The QIA expressionist TM, QIAGEN, Valencia, CA, USA). Column washing buffers (50 mM NaH_2_PO_4_, 300 mM NaCl, and imidazole stepwise in increasing concentrations (10, 30, 60, and 100 mM); pH 8) were used to wash the column sequentially. After washing steps, bound proteins were eluted with elution buffer (50 mM NaH_2_PO_4_, 300 mM NaCl 250 mM imidazole, pH 7.6). The eluted fractions were pooled, concentrated by ultrafiltration (Amicon), and dialyzed against PBS. The protein concentrations were determined by a BCA assay kit (Pierce, Thermo Scientific, Waltham, MA, USA). The endotoxin level of the purified proteins was quantified by QCL-1000 Chromogenic Limulus amoebocyte lysate test according to the manufacturer protocol (BioWhittaker, Walkersville, MD, USA), which showed less than 25 endotoxin units per 50 µg of the purified protein (i.e., acceptable for immunization). The purified rCore and rNS3 proteins were used as an immunogen in combination with purified rNT (gp96) protein and CpG ODN as an adjuvant in mice immunization procedures. The same proteins were also used as coating Ag for ELISA and stimulation of the splenocytes in cytokine.

#### 2.1.3. Mice and Vaccination Regimens

Three different prime-boost regimens were carried out in ten groups of 6-week-old female BALB/c mice (*n* = 15 per group) obtained from the breeding stock maintained at Pasteur Institute of Iran. All experiments were performed according to the guidelines approved by the Ethical Committee of School of Public Health, Tehran University of Medical Sciences (Code of Ethics: 95-02-27-31344). Group1 (G1) was immunized twice with 10 μg rCore, and 10 μg rNS3 formulated with 10 μg rNT (gp96) adjuvant for both prime and boost immunizations (hereafter; protein/protein + rNT (gp96)). Group2 (G2) was immunized three times with 80 μg of pcDNA-Core-NS3-NT (gp96) plasmid in PBS for both prime and boost immunizations (hereafter; DNA-NT (gp96)/DNA-NT (gp96)). Group3 (G3) was vaccinated with 100 μg of pcDNA-Core-NS3-NT (gp96) plasmid in PBS for prime but twice with 10 μg rCore and 10 μg rNS3 formulated with 10 μg rNT (gp96) for boost immunizations (hereafter; DNA-NT (gp96)/protein + rNT (gp96)). Group 4 (G4) received three times PBS as control. Group 5 (G5) was vaccinated three times with 80 μg of pcDNA-Core-NS3 plasmid in PBS for both prime and boost immunizations (hereafter; DNA/DNA). Group 6A (G6A) was vaccinated with 10 μg rCore and 10 μg rNS3 in combination with 40 μg CpG ODN adjuvant (5/-TCC ATG ACG TTC CTG ACG TT-3/) for both prime and boost immunizations (hereafter; protein/protein + CpG). Group 6B (G6B) was vaccinated with 10 μg rCore and 10 μg rNS3 with 40 μg non-CpG (5/-TCC AGG ACT TCT CTC AGG TT-3/) for both prime and boost immunizations (hereafter; protein/protein + non-CpG). Group7 (G7) was immunized three times with 80 μg of empty pcDNA3.1(+) plasmid in PBS for both prime and boost immunizations (hereafter; control DNA/DNA). Group8 (G8) was immunized twice with 10 μg rNT (gp96) for both prime and boost immunizations (hereafter; rNT (gp96)/ rNT (gp96)). Group9 (G9) was immunized twice with 10 μg rCore and 10 μg rNS3 for both and boost immunizations (hereafter, control protein/protein). All groups were vaccinated intramuscularly in the tibialis anterior muscle at a three-week interval, as summarized in Table 2.

### 2.2. Cytokine Assay

Three mice in each group were euthanized randomly at W3 and W11. The ACK (ammonium-chloride-potassium) lysis buffer (NH4Cl 0.15 mM, KHCO_3_ 1 mM and Na_2_ EDTA 0.1 mM; pH 7.2) was used to lyse the erythrocytes. After washing in DMEM, up to 2 × 10^6^ splenocytes were seeded per well in 96-well plates in complete DMEM (5% FCS, 0.1% l-glutamine, 1% HEPES, 0.1% 2ME, and 0.1% gentamicin) and stimulated with rCore and rNS3 at a concentration of 5 μg/mL at 37 °C in 5% CO_2_. Cells with no treatment (medium alone) and cells incubated with concanavalin A (Con A, 5 μg/mL) were used as negative and positive controls, respectively. The cell culture supernatants were harvested after 72 h for IL-4 assay and 96 h for IFN-γ assay. The levels of cytokines were detected using a sandwich ELISA according to the manufacturer’s protocol (R & D, Quantikine@ ELISA, Minneapolis, MN, USA). All assays were performed in duplicates. The lower detection limit of both IFN-γ and IL-4 was 2 pg/mL, and the concentration was calculated according to the standard curve.

### 2.3. Antigen–Specific Antibody Responses

To assess the humoral immune responses, blood samples were taken from each mouse’s orbital plexus before immunization at W3 and W11. The Core-NS3-specific levels of total IgG, IgG1, IgG2a, and the ratio of IgG2a/IgG1 isotype responses were measured by ELISA as described previously [31]. Briefly, 96-well Maxisorp plates (Greiner, Germany) were coated with 100 μL (5 μg/mL in PBS) of the rCore and rNS3 overnight at 4 °C. The plate was washed and incubated with blocking buffer (5% skim milk in PBS) for 2 h at 37 °C. After washing steps, 100 μL pooled sera of each group was added (1:300) to the plates in duplicate and incubated at 37 °C for 2 h. Subsequently, the HPR-conjugated goat anti-mouse IgG, IgG1, or IgG2a (1:6000; Southern Biotech Canada) was added to each plate and incubated for 2 h at 37 °C. After the last washing, the plates were incubated for 30 min with 100 μL of TMB as substrate, and the absorbance was measured at 450 nm.

### 2.4. Statistical Analysis

GraphPad Prism version 8.0.2 statistical software (GraphPad Software, San Diego, CA, USA) was carried out to evaluate statistical analysis. The significance of experimental differences was analyzed using one-way ANOVA (Tukey’s multiple comparison test) and Student’s *t*-test. A *p*-value < 0.05 was measured statistically significant. All data are presented as mean ± SEM.

## 3. Results

### 3.1. Analyses on Expression of the Recombinant Core, NS3, and NT (gp96) Proteins

**Figure 2:**
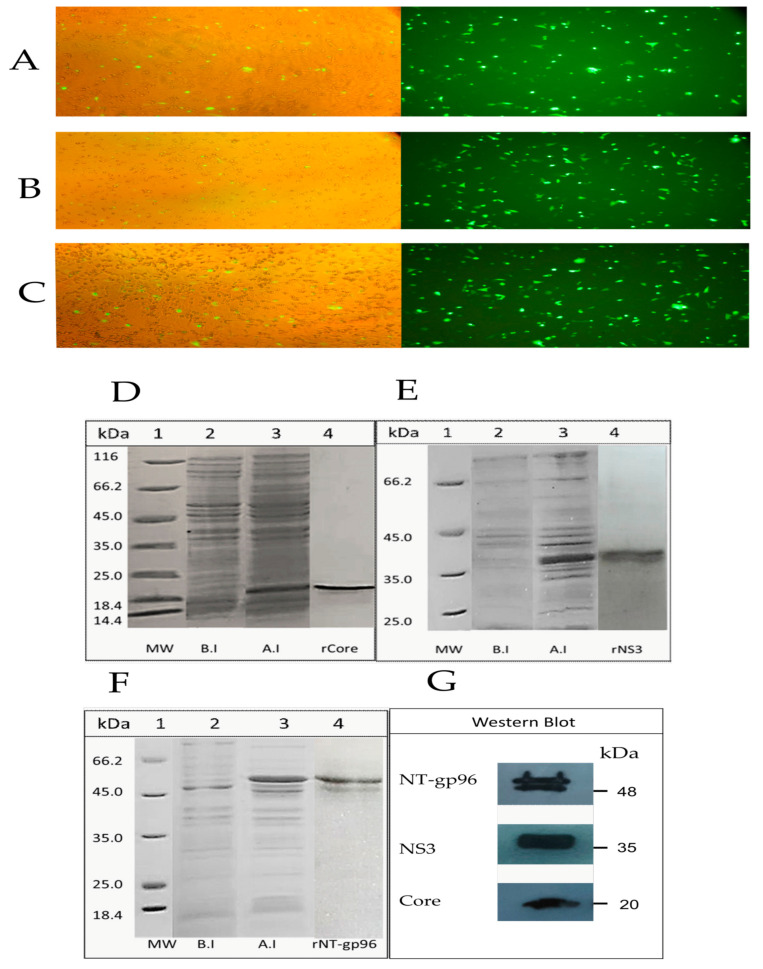
In vitro expression of Core-NS3-EGFP and Core-NS3-NT (gp96)-EGFP in COS-7 cells. (**A**–**C**) Recombinant pEGFP-Core-NS3, pEGFP-Core-NS3-NT (gp96), and pEGFP-N3 plasmids were transiently transfected into COS-7 cells using polyethyleneimine. GFP expression of the transfected cells (before and after glinting fluorescence) with pEGFP-N3 (as positive control), pEGFP-Core-NS3, and pEGFP-Core-NS3-NT (gp96), respectively. SDS-PAGE analysis of rCore (**D**), rNS3 (**E**), and rNT (gp96) (**F**) proteins: Molecular weight markers (lane 1), bacterial extract before induction by IPTG (lane 2), crude bacterial lysate 4 h after induction (lane 3) and recombinant proteins purified from bacterial lysates by affinity chromatography using Ni-NTA matrix (lane 4). (**G**) Western blot analysis of purified recombinant proteins using anti-His mAb and goat anti-mouse IgG antibodies.

As shown in Figure 2A,B, Core-NS3 and Core-NS3-NT (gp96) were efficiently expressed 24 h post-transfection in COS-7 cells, and the antigen expression levels of the two constructs were similar. GFP expression of the positive control was also observed (Figure 2C).

The expression of the core, NS3, and NT (gp96) proteins in *E. coli* was analyzed by SDS-PAGE (Figure 2D–F) and western blotting using anti-His mAb and goat anti-mouse IgG antibodies (Figure 2G). SDS-PAGE showed single bands with the molecular mass of 20 KDa and 38 KDa corresponding to rCore and rNS3 proteins, respectively (Figure 2D,E). SDS-PAGE analysis indicated a prominent band with a molecular mass of 51 KDa corresponding to rNT (gp96) (Figure 2F). SDS-PAGE of this protein after purification using Ni-NTA resin and also western blot analysis revealed a faint second band slightly below the prominent band, which might be a cleavage product (Figure 2G).

### 3.2. Protein/Protein + rNT (gp96) Vaccination Induced Strong and Long-Term Th1 Cytokine Profile

**Figure 3:**
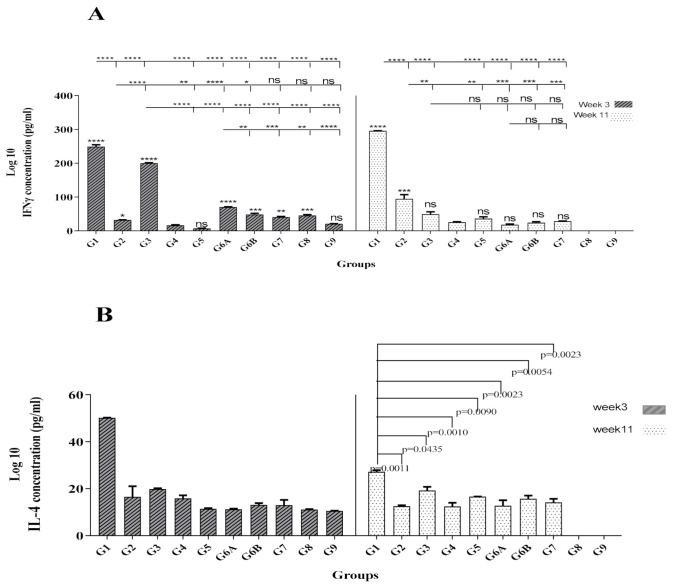
Analysis of cytokine profile in vaccinated and control mice groups. Splenocytes of mice were isolated at 3 and 11 weeks after the last immunization and stimulated by either rCore-rNS3 proteins (5 μg/mL) or Con A (5 μg/mL) for three and five days. Subsequently, IFN-γ (**panel A**) and IL-4 (**panel B**) levels in cell supernatants were measured by ELISA. All assays were performed in duplicate and at least three mice. Bars represent mean values, and SEM are expressed in pg/mL. Statistical analyses were done by one-way ANOVA (*p* < 0.05 denoted as *, *p* < 0.01 denoted as **, *p* < 0.001 denoted as ***, *p* < 0.0001 denoted as **** and non-significant denoted as ns). The asterisks indicated directly above the bars demonstrate statistically significant differences between the vaccinated groups and the control group of mice (G4).

The IFN-γ production level (as an indicator of Th1-oriented immunity) was measured using the supernatant of Ag–stimulated splenocytes of all mice groups at W3 and W11. As shown in Figure 3A, G1 groups indicated significantly higher levels of IFN-γ at W3 and W11 (*p* < 0.0001). Our results also revealed a significantly higher level of IFN-γ in G3 compared to G2, G5, G6A, and all control groups at W3 (*p* < 0.0001), but the same pattern was not observed for this group at W11. Moreover, in contrast to G3 and G6A, other vaccinated groups G1, G2, and G5 indicated an increase in IFN-γ level up to 1.2, 2.93, and 5.32-fold, respectively, at W11 (Figure 3A). We further evaluated whether immunized mice produced Th2-associated cytokine IL-4. There was a significant difference between G1 and other vaccinated and control groups in IL-4 level at W3 (*p* < 0.0001, Figure 3B). Although G1 indicated a clear decrease in IL-4 production at W11, this group still showed a significant difference with other mice groups (*p* < 0.05, *p* < 0.01, *p* < 0.001, Figure 3B). Accordingly, at W3 and W11, the IFN-γ/IL-4 ratios for vaccinated groups (except for G5 at W3) indicated a bias toward the Th1 response (data not shown). At W3, the highest ratio belonged to the G3 group, but it decreased significantly at W11. In contrast, the G1 group showed increased levels of IFN-γ/IL-4 ratio at W11, indicating the highest long-term ratio. Overall, results demonstrated the long-term potency of protein/protein + rNT (gp96) vaccine formulation in enhancing the Th1-type immune responses.

### 3.3. Formulation of HCV Core-NS3 Proteins with rNT(gp96) Elicited Th1-Phenotype Antibody Responses

**Figure 4:**
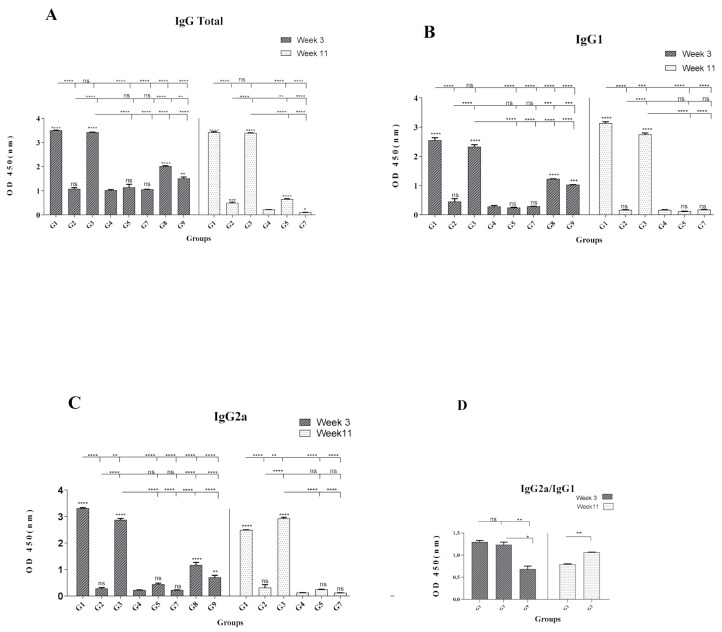
Antigen-specific total IgG, IgG1, and IgG2a antibody responses in vaccinated and control mice groups. Sera were obtained from each group’s mice and pooled (*n* = 8) and tested for anti-rCore-rNS3 proteins at W3 and W11. Panel (**A**) shows specific total IgG levels, panel (**B**) indicates specific IgG1, panel (**C**) shows specific IgG2a, and panel (**D**) indicates the IgG2a/IgG1 ratio. All samples were measured in duplicate. Data are represented as mean ± SEM. Statistical analyses were done by one-way ANOVA. (*p* < 0.05 denoted as *, *p* < 0.01 denoted as **, *p* < 0.001 denoted as ***, *p* < 0.0001 denoted as **** and ns denoted as non-significant). The asterisks shown directly above the bars indicate statistically significant differences between vaccinated groups and mice’s control group (G4).

Although antibodies raised against HCV core and NS3 proteins, have no neutralization value, but to gain insights about the potential of rNT (gp96) as an adjuvant for induction of humoral responses, the level of specific Abs against rCore-rNS3 proteins in the sera of the corresponding mice groups were evaluated. As shown in Figure 4A, there was no significant difference in total IgG levels between G1 and G3, while these vaccinated groups indicated a significantly higher level of total IgG compared to other vaccinated (G2, G5) and control groups at W3 and W11. At W3 and W11, no significant difference was observed for IgG1 level between G1 with G3 group (Figure 4B). At W3 and W11, G1 induced IgG2a levels, which was significantly higher than that of the G3 group at W3 (*p* < 0.01, *p* < 0.001, Figure 4C). In contrast, at W11, an increase of IgG2a level in G3 led to a significantly higher level of this isotype in G3 compared to G1 (*p* < 0.01, Figure 4C). The lowest level of total IgG, IgG1, and IgG2a was observed in G2 and G5 groups immunized with DNA-NT (gp96)/DNA-NT (gp96) and DNA/DNA, respectively (Figure 4A–C). At W3, the IgG2a/IgG1 ratio in G1 and G3 groups showed a shift toward Th1 immune responses; however, at W11, this ratio decreased to <1 for G1 and was about 1 for G3, suggesting a shift toward a Th2 response and a more balanced Th1-Th2 phenotype, respectively (Figure 4D).

## 4. Discussion

The present study was designed to evaluate the capacity of recombinant protein NT (gp96) as an adjuvant to enhance immune responses in the formulation of HCV rCore and rNS3 proteins as an immunogen. To our knowledge, this is the first report on the utilization of recombinant gp96 protein as an adjuvant to develop a candidate HCV protein vaccine. In addition, the efficacy of three immunization regimens, including DNA/DNA, DNA/protein, and protein/protein for HCV rCore and rNS3 proteins, was compared in the BALB/c mice model. Our results indicated that immunization with a protein vaccine formulated with rNT (gp96) protein as an adjuvant in a homologues protein/protein + rNT (gp96) strategy (G1) could efficiently induce short and long-term Th1-type cytokines compared to other immunization regimens (protein/protein + CpG, DNA/protein, and DNA/DNA). Moreover, results of the present study indicated that NT (gp96) as a recombinant protein adjuvant along with HCV proteins (core/NS3) could induce more potent immune responses than the previously reported vaccine formulations comprising NT (gp96) fragment fused to HCV polytope DNA (PT) (PT-NT (gp96)) [27].

Considering the importance of Th1 cytokine production for protection from HCV infection and prevention of chronicity, we evaluated our vaccine formulations for induction of these cytokines by cytokine assay. Of note, immunized mice in G1 not only elicited significantly higher levels of IFN-γ than other vaccinated groups at W3 and W11 but also showed an increase of IFN-γ (1/2 fold) production and a concomitant decrease in IL-4 (1/8 fold) at W11 compared to W3 (Figure 3A,B). These results showed a stronger tendency of Th1-type response in mice immunized with protein vaccine formulated with gp96 (G1) as well as the enhancement of this response in the long-term compared to other groups. In fact, both G1 and G2 immunized mice showed similar long-term levels of strong Th1-type responses at W11 compared to other groups (Figure 3A,B) that imply the positive effect of the NT (gp96) adjuvant in their formulation. Indeed, it is suggested that NT (gp96) might enhance the take-up and Ag processing by APCs and their activation, resulting in the release of proinflammatory cytokines by these cells that finally ends up with a protective Th1 response in HCV infection [32]. Therefore, co-administration of recombinant NT (gp96) protein with rHCV proteins might be a promising approach in the formulation of HCV subunit vaccine candidates for induction of higher levels of Th1 polarized cytokine responses.

As shown in Figure 4, the G1 group indicated a significant increase in total IgG, IgG1, and IgG2a titers and IgG1/IgG2a ratio with Th1 polarization characteristics compared to the control groups. Consistent with our observation, formulation of Hepatitis B virus (HBV) Ags (HBc and HBs proteins) with rNT (gp96) also resulted in the induction of significantly higher total IgG, IgG1, and IgG2a levels and IgG1/IgG2a ratio with Th1 polarization characteristics. Our results are also in agreement with another report on immunization by HPV16E7 protein formulated with gp96, which showed Th1-biased mixed Th1/Th2 immune responses in mice immunized by this immunogen [33]. It might be worth mentioning that the presence of Th2 type responses might not prevent the induction of strong CTL and cytokines, as previously shown for anti-tumor responses against tumor specific Ags [34]. Our results from G2 and G5 groups did not show a potent antibody response (Figure 4A–C). The reasons for these observations might be partly due to the low amount of antigens produced by DNA vaccines [21,35] or the inefficiency of HCV core when administered as a DNA immunogen [36]. Therefore, although antibodies raised against HCV core and NS3 proteins have no neutralization value, but our results indicated the potency of rNT (gp96) as an adjuvant for induction of high-level humoral responses, which might be considered in the formulation of HCV E2 protein (to induce neutralizing Abs).

It should be noted that the main “aim” of the present study was studying the potential of gp96 in combination with HCV core/NS3 Ags for induction of Th1-type cytokines as a “proof of concept” with no claim for protection from HCV infection by this immunogenic formulation. Nevertheless, for the next step, “pre-clinical studies,” a proper animal model and challenging studies might be considered.

## 5. Conclusions

Overall, in this study, we provided the first report on the application of rNT (gp96) as an adjuvant for the induction of more potent immune responses against rCore and rNS3 Ags in a protein (subunit) vaccine candidate. Our preliminary findings suggested rNT (gp96) as a promising adjuvant for vaccination purposes to HCV and demonstrated that immunization with homologous protein/protein-rNT (gp96) strategy was capable of inducing the highest level of Th1-type cytokine. Despite recognizing no neutralization value for anti- Core/NS3 Abs, but our results indicated the potency of rNT (gp96) as an adjuvant for induction of high-level humoral responses, which might be considered in the formulation of HCV E2 protein (to induce neutralizing Abs). Therefore, collectively, the co-administration of recombinant NT (gp96) protein with rHCV proteins might be a promising approach in the formulation of HCV subunit vaccine candidates for the induction of high levels of Th1 cytokines and humoral responses.

## Figures and Tables

**Table 1:** List and sequence of the primers.

Characteristics	Sequence
Forward Kgp1 with *Not*I site (underlined)	5′ATA TGC GGC CGC GAA GAT GAC GTT G3′
Reverse Kgp2 with stop codon (bold) and *Xba*I site (underlined)	5′GGG CTC TCT AGA **TTA** TTT GTA GAA GGC TTT G3′
Forward KZ1 with *EcoR*I site (underlined)	5′TCC GAA TTC GAA GAT GAC GTT GAA GTG3′
Reverse KZ2 with *Apa*I site (underlined)	5′CTC GGG CCC ATTTT GTA GAA GGC3′
Reverse KZ3 with stop codon (bold) and *Apa*I site (underlined)	5′CTC GGG CCC **CTA** TTT GTA GAA GGC3′
Forward ZC1 with *Nco*I and *Nhe*I sites (underlined)	5′ATC GAT GCT AGC GAG CTC ACC A3′
Reverse ZC2 with *BamH* I site (underlined)	5′CAA CAG CGG ACC GGA TCC AAG C3′
Forward ZN1 with *Nhe* I and *BamH* I sites (underlined)	5′CTT GCT AGC GGA TCC GGT CCG CTG TTG3′
Reverse ZN2 with *Not*I sites (underlined)	5′GTG GCG GCC GC**T TAT** CTA GAC TGC AG3′

**Table 2:** Immunization schedule in different mice groups.

Groups	Prime 1st Week	Boost 4th Week	Boost 7th Week	Modeling
G1		Core-NS3 + NT (gp96)	Core-NS3 + NT (gp96)	Protein/protein
G2	pcDNA-Core-NS3-NT (gp96)	pcDNA-Core-NS3-NT (gp96)	pcDNA-Core-NS3-NT (gp96)	DNA/DNA
G3	pcDNA-Core-NS3-NT (gp96)	Core-NS3 + NT (gp96)	Core-NS3 + NT (gp96)	DNA/protein
G4	PBS	PBS	PBS	Control
G5	pcDNA-Core-NS3	pcDNA-Core-NS3	pcDNA-Core-NS3	Control
G6A		Core-NS3 + CpG ODN	Core-NS3 + CpG ODN	protein/protein
G6B		Core-NS3 + non-CpG	Core-NS3 + non-CpG	Control
G7	pcDNA3.1(+)	pcDNA3.1(+)	pcDNA3.1(+)	
G8		NT (gp96) protein	NT (gp96) protein	Control
G9		Core-Ns3 protein	Core-Ns3 protein	Control

## Data Availability

Data can be requested by writing to the authors.
